# Sintilimab combined with anlotinib as first-line treatment for advanced sarcomatoid carcinoma of head and neck: a case report and literature review

**DOI:** 10.3389/fonc.2024.1362160

**Published:** 2024-04-24

**Authors:** Lei Wang, Yingyu Huang, Xin Sun

**Affiliations:** ^1^ Graduate School of Clinical Medicine, Bengbu Medical University, Bengbu, Anhui, China; ^2^ Cancer Center, Department of Medical Oncology, Zhejiang Provincial People’s Hospital, Affiliated People’s Hospital, Hangzhou Medical College, Hangzhou, Zhejiang, China; ^3^ Graduate School of Hangzhou Normal University, Hangzhou, Zhejiang, China

**Keywords:** sarcomatoid carcinoma, head and neck, anlotinib, sintilimab, immune checkpoint inhibitors

## Abstract

Sarcomatoid carcinoma (SC) is a rare, complex, aggressive tumor that spreads rapidly, is highly malignant, and has metastasized. Surgical resection is the primary treatment, and it usually occurs in the lungs and kidneys but rarely in the neck. Patients with advanced sarcomatoid carcinoma (SC) of the head and neck (HN) have a poor progonsis. In recent years, immune checkpoint inhibitors (ICIs) have been established as treatments for many solid tumors; however, the effectiveness of ICIs in treating SC of HN is still little recognized. We report a case study of a middle-aged woman with primary sarcomatoid carcinoma of the neck. She developed sarcomatoid carcinoma of the contralateral neck 7 months after the first surgical treatment. Subsequently, disease recurrence and metastasis occurred 8 months after the second surgery. The patient did not receive any treatment after both surgeries. The tumor showed high programmed death-ligand 1 (PD-L1) expression, with a combined positive score (CPS): 95. The patient’s response to treatment was assessed as partial remission (PR) after 2 cycles of anlotinib combined with sintilimab. The patient has survived for over 2 years and remains in PR status, despite experiencing grade 2 hypothyroidism as an adverse event during treatment. The case highlights the efficacy and safety of anlotinib and sintilimab as a first-line treatment.

## Introduction

Sarcomatoid carcinoma (SC) is a rare, complex, and highly malignant tumor that presents biphasically in terms of epithelial carcinoma and mesenchymal-like sarcoma components. Since its first report in 1864, SC has been documented in various organs including the lungs, uterus, kidneys, bladder, colon, liver, gallbladder, and skin (1–8). Among all head and neck malignancies, the incidence of sarcomatoid carcinoma was 0.57% (9). Most sarcomatoid carcinoma (SC) of the head and neck (HN) originated in the larynx, nasal cavity, and maxillary sinuses, and rarely in the neck. Due to the rarity SC of HN, limited information can be found in previous reports, which mainly consist of case studies or small retrospective studies. These studies have shown a median survival time of 16 months for patients with SC of HN (10).

Patients with SC of HN often develop lymph node metastasis, which contributes to their poorer prognosis. Currently, there are no specific clinical treatment guidelines available; surgical resection has been shown to be an effective option for early stage SC of HN (9). However, even after radical surgery in the early stage, recurrence and metastasis remain significant concerns (11). Most patients with SC of HN are diagnosed at advanced or distantly metastatic, missing the opportunity for surgery. The majority of these patients received palliative radiotherapy and chemotherapy, resulting in poor prognosis. Therefore, there is an urgent need to develop new effective therapeutic options for this condition. In recent years, immune checkpoint inhibitors (ICIs) have shown a positive response in various malignancies. The U.S. Food and Drug Administration has approved pembrolizumab, a programmed death-1 (PD-1) inhibitor, as a first-line treatment for recurrent or metastatic head and neck squamous cell carcinoma (HNSCC) where tumor cells expressing programmed death-ligand 1(PD-L1) with combined positive score CPS≥1 (12). Babacan et al. found that over 90% of patients with Lung sarcomatoid carcinoma (LSC) had PD-L1 expression level exceeding 1%, and the level of PD-L1 expression was closely correlated with the efficacy of ICIs (13). Patients with LSC who received ICIs showed significantly better outcomes compared to those underwent traditional radiotherapy and chemotherapy (14). Besides LSC, high expression of PD-L1 has been found in other types of SC (15).

Sintilimab, a recombinant fully human immunoglobulin G4 (IgG4)-type anti-programmed cell death receptor-1 monoclonal antibody (16), has shown good objective response rate (ORR) and overall survival (OS) in advanced HNSCC (17). Tyrosine kinase inhibitor (TKI) have also demonstrated positive responses in various malignancies. Several TKIs targeting head and neck malignancies are currently under investigation, with the hope of benefiting from clinical trials (18). Anlotinib is a multi-targeted TKI that inhibits tumor angiogenesis and proliferation (19). It is approved by the National Medical Products Administration for the treatment of advanced non-small cell lung cancer and soft tissue sarcomas. Due to the low incidence of SC of HN, the use of ICIs in combination with targeted therapy has been reported to be restricted. Maybe SC of HN has a positive response to PD-1/PD-L1 inhibitors in combination with targeted therapy.

Here, we report a case of advanced primary sarcomatoid carcinoma of the neck with multiple metastases showing high expression of PD-L1 (CPS: 95). This is the first reported case demonstrating the efficacy of anlotinib in combination with sintilimab as a first-line treatment for SC of HN.

## Case description

A 66-year-old female patient with a 4.5 cm subcutaneous lump on her left side of the neck arrived at a local hospital in December 2021. She reported no swelling or pain, and denied any family history of tumors and had no obvious medical history. Then, the lump was surgically removed under local anesthesia. The pathology report revealed that the tumor exhibited infiltrative growth, was situated in the dermis and subcutis, and consisted of heterogeneous cells with vacuolated nuclei and eosinophilic nucleoli, as well as easily visible mitotic pictures. The immunohistochemical (IHC) staining results were as follows: CK(pan)(+), Ki-67(70%+), P63 (–), MelanA (–), S100 (–), SOX10 (–), CD35 (–), EBER (–), ALK (–), CD20, lymphocyte(+), CD30lymphocyte(+), Desmin (–), CD34 (–), EMA (–), SMA (–), ERG (–), CK7(+), CK5/6 (–), CK8(+). PD-L1 was expressed the CPS was 30. SC of the left neck was considered according to the aforementioned results combined with IHC data. The patient did not receive any treatment after the surgery.

**Figure 1:**
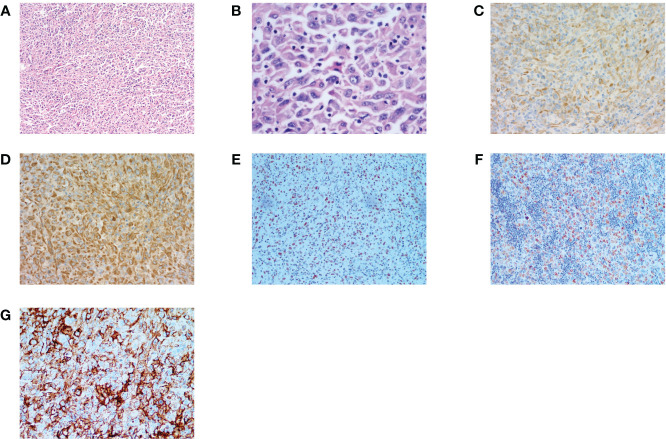
Histopathology and immunohistochemistry (IHC) of HNSC. **(A, B)** H&E stain, original magnification ×100,×400. **(C)** IHC CK(pan)(+) original magnification ×200. **(D)** IHC Vimentin(+), original magnification ×200. **(E)** IHC GATA3(+) original magnification ×20. **(F)** IHC Melan(+) original magnification ×20. **(G)** PD-L1 IHC (Dako22C3), original magnification ×40. Combined Positive Score [CPS]: 95. (CPS was defined as the number of PD-L1 stained cells (tumor cells, lymphocytes, macrophages) divided by the number of all tumor cells and multiplied by 100.).

In August 2022, 7 months after surgery, the patient developed a 4 cm subcutaneous nodule on the right side of her neck. In November 2021, the patient returned to the local hospital for an ultrasound examination of the neck, which revealed enlargement of the left lymph nodes. Considering that the patient had a history of SC on the left neck, an “ Enlargement Excision Procedure of the tumor in the right neck + clearance procedure of the left lymph nodes” was performed on November 23, 2022. The pathological results showed a large number of tumor cells were diffusely distributed in the dermis and subcutis with infiltrative growth. The tumor cells were heterogeneous, and the nuclear schizophrenia was easy to see with a large number of chronic inflammatory cells infiltrating the interstitium, and some areas of necrosis. Additionally, the left neck lymph node specimen revealed a malignant tumor with lymph node metastasis. IHC staining showed: GATA-3(+), INI-1(+), CK(pan)(+), SOX10 (–), Vimentin(+), MelanA(+), TTF-1 (–). PD-L1 was strongly expressed with a CPS was 95 (**Figure 1**). SC of the right side of the neck with lymph node metastasis was diagnosed on the basis of pathological and IHC findings. Despite the diagnosis, the patient did not pursue further treatment after the second operation.

**Figure 2:**
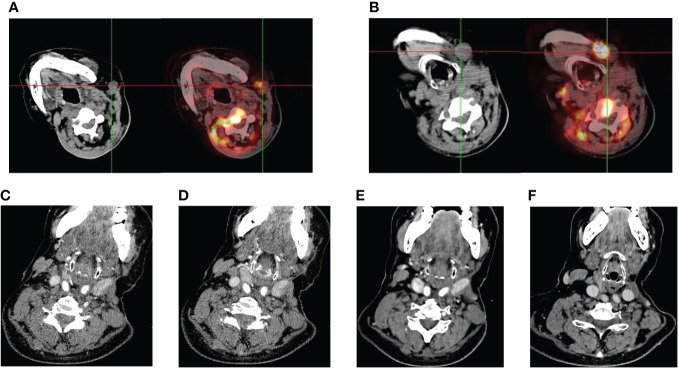
Imaging performance. **(A, B)** Eight months after the second surgery, the patient’s PET/CT examination suggested tumor recurrence and metastasis with multiple lymph node metastases in the left side of the neck. **(C–F)** continuous shrinkage of the tumor lesion and metastatic lymph nodes following sintilimab combined with anlotinib treatment in October 2023, December 2023, respectively.

**Figure 3:**
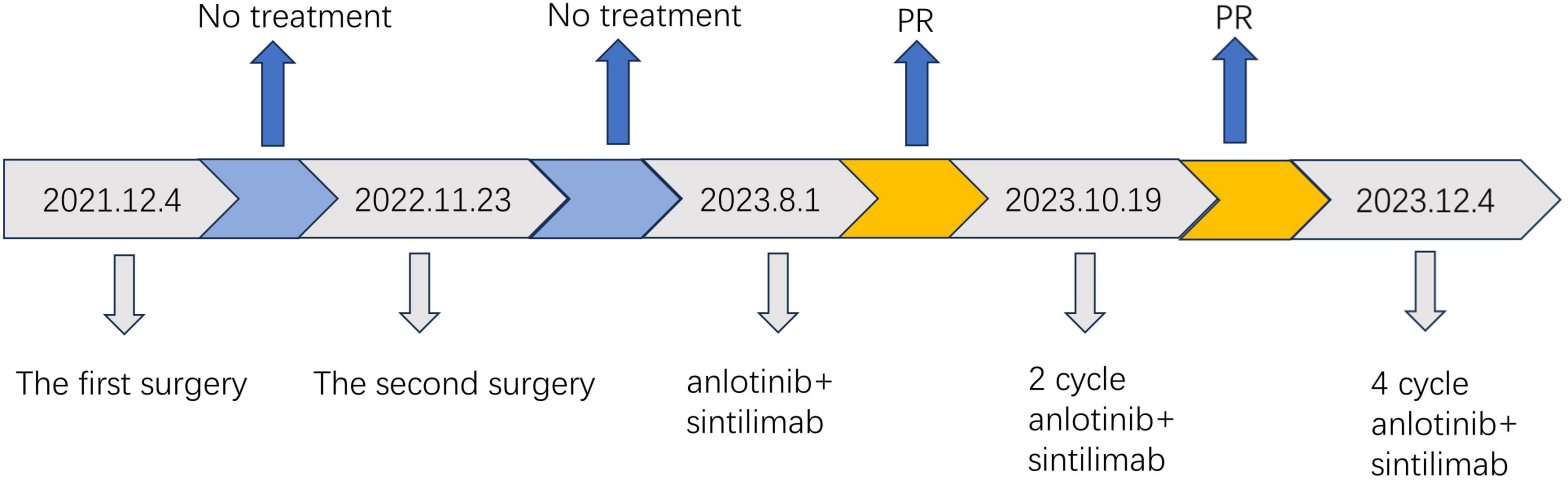
Timeline of treatment process.

In June 2023, the patient came back to the hospital with self-conscious neck pain as well as limitation of movement. Enhanced magnetic resonance imaging (MRI) of the cervical spine showed abnormal signals in the C3 vertebrae and intervertebral foramina on both sides. In July 2023, positron emission tomography (PET)/CT suggested multiple lymph node metastases in the left neck and axillary fossa; and inhomogeneous increase in 18F-fluorodeoxyglucose (18F-FDG) metabolism in the C2-C4 vertebrae and their surrounding soft tissues (**Figures 2A, B**). We recommended chemotherapy to the patient, but the patient strongly refused due to concerns about its side effects and requested an immune checkpoint inhibitor. Previous reported cases of other types of SC, the PD-1 inhibitor combined with anilotinib regimen had good efficacy. Given the patient’s high PD-L1 expression, after full discussion with the patient and her family, they agreed to use the sintilimab in combination with anilotinib regimen. The PD-1 inhibitor sintilimab 200 mg q3w and anlotinib 8mg (2 weeks on/1 week off) was implemented on August 1, 2023. After 2 cycles of targeted and immunotherapy, on September 13, 2024, the patient’s thyroid function was abnormal, and she was evaluated by an endocrinologist to confirm the diagnosis of drug-induced hypothyroidism, and was given eugenol 50ug per day as replacement therapy. We temporarily internpted sintilimab and anlotinib. On October 18, 2023, an enhanced Computed tomography (CT) examination of the patient’s neck suggested significant tumor reduction (**Figures 2C, D**), and the function of the thyroid was better than before. According to the Response Evaluation Criteria in Solid Tumors (RECIST) criteria, the efficacy was evaluated as partial remission (PR). Due to the significant anti-tumor therapy, we continued with the original regimen. After 4 cycles, the tumor was further reduced and the clinical efficacy was evaluated PR (**Figures 2E, F**). The patient is continuing treatment. The treatment timeline is shown in **Figure 3**.

## Discussion

SC is a rare aggressive cancer that can occur in various organs, with the lungs and kidneys being the most common primary sites. SC of HN has a low incidence and is considered to be a monoclonal dedifferentiated form of HNSCC with a high degree of malignancy and poor prognosis. Several studies have indicated that SC patients are mainly male, and the age of onset is around 60 years old. The etiology and the pathogenesis of the disease are still unclear; however, current evidence suggests that smoking, alcoholism, and exposure to radiation may contribute to its development (12–14). There are no specific clinical manifestations in the early stages of SC of HN, and no specific changes on relevant radiological images. Histopathology and IHC are the gold standard for diagnosing the disease, which microscopically shows a biphasic manifestation of coexistence of epithelioid carcinoma component and mesenchymal sarcoma component. IHC analysis reveals positivity for cytokeratin (CK) in the epithelial component and vimentin in the sarcomatoid component, or a combination of both markers (11). Our case is an elderly female with nonspecific clinical symptoms ultimately diagnosed with a classic sarcomatoid carcinoma of the head and neck based on immunohistochemical findings.

SC of HN is much more malignant than squamous cell carcinoma. It has a tendency for early lymph node metastasis, rapid progression, and a poor prognosis. The main factors influencing the prognosis of SC of HN include tumor location, tumor size, tumor stage, and lymph node metastasis (9, 10, 15). Although surgical resection is currently the best treatment, it is susceptible to metastasis and recurrence even following early intervention with surgical resection. Naijen et al. conducted a study on 78 patients with SC of HN, revealing that 66% of 64 patients who underwent surgery experienced recurrence, the average survival period post-recurrence ranged from 2 to 5 months (16). Our patient developed sarcomatoid carcinoma of the contralateral neck 7 months after the first surgical treatment, and disease recurrence and metastasis 8 months after the second surgery, without adjuvant radiotherapy after both surgeries, indicating that sarcomatoid carcinoma of the head and neck is aggressive, highly malignant, and easily metastasized. Controversy still exists as to whether preoperative or postoperative chemotherapy or radiotherapy can reduce tumor recurrence and metastasis. In a study on laryngeal sarcomatoid carcinoma by Dubal et al. (17), it was observed that the disease-specific survival (DSS) at 5 years after surgery was found to be 84.1%, which was much higher than the DSS at 5 years for non-surgical treatments (57.1%), and it also found that the addition of adjuvant radiotherapy did not significantly improve the survival rate. In another report on hypopharyngeal sarcomatoid carcinoma, researchers discovered that adjuvant therapy after surgery did not result in an improvement in DSS (15). However, both radiotherapy and chemotherapy are still effective in the treatment of local and distant metastases of head and neck tumors. Further clinical studies are needed to ascertain the effectiveness of adjuvant radiotherapy and chemotherapy in SC.

Several studies have found that PD-L1 is highly expressed in patients with SC. Approximately 72% in LSC (18), 50% in sarcomatoid renal cell carcinoma (19) and about 75% in SC of HN (20). These results suggest that SC may be sensitive to immunotherapy. So far, there are no guidelines recommending immunotherapy for advanced SC, mainly case reports and retrospective studies. We searched for previously published papers on ICIs for the treatment of various SC and summarized them in **Table 1**. Whether it is single immunotherapy or immunotherapy combined with amlotinib, progress free survival (PFS) and overall survival (OS) are satisfying. The higher the patient’s PD-L1 expression, the better the effect.

**Table 1:** Previous reports of SC treated with ICIs.

Author	Report type	Number	Tumor types	Regimen	Efficacy	Predictive biomarkers
Gounant, V.,ect (21)	case	1	LSC	Nivolumab	DOR>10months.	PD-L1 expression in tumor was 80%.
Salati,M., ect (22).	case	1	LSC	Nivolumab	DOR>28months.	PD-L1 TPS=50%
Kotlowska,M.P., etc. (23)	case	1	LSC	ICIs	9 months PFS.	PD-L1 expression was 95%.
Roesel, C.,etc. (24)	case	2	LSC	Nivolumab	Case1: Disease was stable at 6 months. Case2: DOR>8months.	PD-L1 expression was 80-100%.
Sukrithan, V.,etc. (25)	case	5	LSC	Pembrolizumab	Case1:23months PFS. Case2:DOR>29 months Case3:DOR>13months. Case4:DOR>11months. Case5:DOR>12months.	Five patients were determined to be positive for PD-L1 (TPS> 75%)
Chen, P., etc. (26)	case	1	LSC	Pembrolizumab	8 months PFS.	PD-L1 TPS=90%
Kong,F., etc. (27)	case	1	LSC	Camrelizumab	20 months PFS.	Patients had high levels of PD-L1 expression.
Jin,C.,etc. (28)	case	1	LSC	Nivolumab	DOR>6months	PD-L1 TPS=90%
Nishino, K.,etc. (29)	case	1	LSC	Pembrolizumab	DOR>7months	PD-L1 expression in 90% of the tumor
Taniguchi, H.,etc. (30)	case	1	LSC	Pembrolizumab	PFS: four cycles	PD‐L1 expression1% on tumor cells
Jiao,Y., etc. (31)	case	1	LSC	Toripalimab	8 months PFS.	PD-L1 TPS=50%
Li,Y.F., etc. (32)	case	1	LSC	Tislelizumab+anlotinib	DOR>20months.	TP53mutation;TMB was 7.7 muts/Mb,
Piao,M. N.,etc. (33)	case	1	LSC	Sintilimab	22 months PFS.	PD-L1 TPS=70%
Sawatari, K.,etc. (34)	case	1	LSC	Atezolizumab	DOR>2 years	PD-L1 expression was negative in the tumor cells
Wan,Y., etc. (35)	case	1	LSC	Camrelizumab	Disease was stable at 18 months.	the expression of PD-L1 as 45%
Xu,L., etc. (36)	cas	1	LSC	Tislelizumab	Disease was stable at 8.5 months.	PD-L1(TPS=60% CPS=90)
Wen,Y., etc. (37)	case	1	LSC	Pembrolizumab+anlotinib	7 months PFS.	PD-L1(TPS=80%); TMB was 14.46 muts/Mb;MSS
Wu,S., etc. (38)	case	1	LSC	Pembrolizumab+anlotinib	OS>45months.	PD-L1(TPS=80%, CPS=95); TMB was 11.52muts/Mb; MSS;TP53 mutation
Raychaudhuri, R., etc. (2)	case	2	SRCC	Nivolumab	Case1:DOR>10months. Case2:DOR>15months.	Both patients had high levels of PD-L1 expression.
Tolay,S., etc. (39)	case	1	SRCC	Nivolumab	DOR>2 years	PD-L1 was strongly expressed
Hino,C., etc. (40)	case	1	SRCC	Nivolumab+ipilimumab	No metastasis for 11months	PD-L1 was strongly expressed
Tomioka, M., etc. (41)	case	1	SRCC	Nivolumab	DOR>20months.	PD-L1(TPS=25% CPS=40)
Fuu,T.,etc. (42)	case	1	SRCC	Nivolumab + ipilimumab	DOR>3 years	PD-L1 TPS≥10%
Zhu,S., etc. (43)	case	1	SUC	Tislelizumab+anlotinib	20 months PFS.	PD-L1 expression (>90% of tumor cells,;TMB was 12.73 mut/Mb
Anraku,T., etc. (44)	case	1	SUC	Nivolumab + Ipilimumab	10 months PFS.	PD-L1 was strongly expressed
Qiu,H., etc. (45)	case	1	SCP	Camrelizumab+anlotinib	DOR>15months.	PDL1(TPS=5%,CPS=7); TMB was 0.15muts/Mb;MSS
Zhang,L., etc. (46)	case	1	SCMS	Camrelizumab	DOR>3months.	PD-L1 expression negative.
Wang,Z., etc. (47)	case	1	SCPT	Toripalimab	DOR>13months	PD-L1 TPS=60%
Present case	case	1	SC of HN	Anlotinib+sintilimab	DOR>4months.	PD-L1 CPS=95; MSS
Qian, X., etc. (48)	observational	21	LSC	ICIs	The median PFS for 9.2 months. The median OS was 22.8months	Six patients (28.5%) PD-L1 TPS >49%
Domblides,C., etc. (49)	observational	37	LSC	ICIs	The median PFS for 4.8months. The ORR was 40.5% (15/37)	PD-L1 H-Score ≥5% had a greater ORR (58.8%).The median TMB was 18mutations/MB。
Wei, J. W., etc. (50)	observational	33	LSC	ICIs	The median PFS for 6.07 months. the ORR was 36.4%	10 patients were determined to be positive for PD-L1.
Zhou,F., etc. (51)	observational	42	LSC	ICIs 4 (11.1%) Chemotherapy +ICIs 37 (88.1%) Chemotherapy + ICIs + bevacizumab 1(2.4%)	The median PFS for 10.3 months. the ORR was 73.8%	PD-L1 TPS<1%,1-49%,and≥50%,the ORR was 33.3%,72.7%,85.7%,mPFS:6,6.7,10.3 months, respectively

LSC, Lung sarcomatoid carcinoma; SRCC, Sarcomatoid renal cell carcinoma; SCP, Sarcomatoid carcinoma of the pancreas; SUC, Sarcomatoid urothelial carcinoma; SCPT, Sarcomatoid carcinoma of the palatine tonsil; SCMS, Sarcomatoid carcinoma of maxillary sinus; PFS, Progress free survival; ORR, Objective response rate; DOR, Duration of response; OS, Overall survival; TPS, Tumor proportion score.

The PD-L1 expression in our patient was remarkably elevated at 95%, potentially explaining the favorable response to sintilimab. Sintilimab, a highly specific fully humanized IgG4 monoclonal antibody, functions by obstructing the binding site of PD-1. It has been shown to be effective in the treatment of a variety of tumors, including HNSCC (52). Anti-angiogenic treatment may also benefit patients with sarcomatoid carcinoma. Kong et al. reported sustained remission in patients with advanced LSC through the application of apatinib in combination with chemotherapy (53). In addition, anlotinib has exhibited positive clinical outcomes in patients with LCS (32, 43). Anlotinib, a new oral tyrosine kinase inhibitor (TKI), targets platelet-derived growth factor receptor, fibroblast growth factor receptor, and vascular endothelial growth factor receptor. It has obtained approval for NSCLC, soft tissue sarcoma, and medullary thyroid carcinoma (54). The clinical benefit for our patients was probably due to the coordinated effect of anti-angiogenic drugs in combination with ICIs. Anlotinib promoted the normalization of tumor vasculature, improved the tumor microenvironment(TME), which changed the immunosuppressive TME into an immunostimulatory TME, and inhibited tumor growth. Additionally, anlotinib can reverse the immunosuppression induced by PD-1 inhibitors, prolonging the time of vascular normalization, and ultimately lead to the elimination of tumors (55). As far as we know, it’s the first case that SC of HC was treated with sintilimab and anlotinib, and the patient sustained PR. Unfortunately, the patient refused genetic testing and we were unable to learn more about the mutation status. A phase II clinical trial is underway (https://classic.clinicaltrials.gov/ct2/show/NCT05265793), enrolling patients with untreated advanced SC to evaluate the effect of first-line karelizumab combination with apatinib treatment, and we look forward to the publication of their results.

Although the efficacy of immunotherapy in SC seems to be satisfactory, accurate biomarkers are still needed to predict the efficacy of ICIs treatment. Current research hotspots mainly include PD-L1 expression, TMB mutations, etc. (**Table 1**). The effect and mechanism of PD-L1 expression in SC are not clear. However, recent studies have shown that PFS was significantly increased in the PD-L1 ≥ 1% group compared with the PD-L1 < 1% group in SC treated with ICIs (14.4 months vs. 2.7 months). In addition, there was a positive correlation between the expression of PD-L1 and the effectiveness of immunotherapy. Higher levels of PD-L1 expression resulting in better efficacy. A retrospective study showed ORR of 33.3%, 72.7%, and 85.7%, and mPFS of 6.0, 6.7, and 10.3 months in patients with PD-L1 TPS <1%, 1-49%, and ≥50%, respectively (51, 56).

TMB is a marker for predicting the efficacy of ICIs. The KEYNOTE-012 trial showed a correlation between TMB values and clinical response in patients with HCSCC. Patients with high TMB had a higher PFS, which was not associated with PD-L1 expression (57). Doomblides, C et al. showed a report on LSC that patients with high TMB had a median OS of 18 months, while the low TMB population had only 1.84 months (49). Other case reports of sarcomatoid carcinoma have also shown a longer survival time with a higher TMB (32, 43, 37, 38).The median value of TMB in patients with sarcomatoid carcinoma of the head and neck was 4.34 (0.71-14.71) muts/Mb, and TMB was significantly higher in patients with advanced stages compared to those with early stages (20). However, the relationship between TMB and immunotherapy in patients with sarcomatoid carcinoma of the head and neck has not been confirmed.

In a small sample, gene sequencing showed that 62% of patients with SC of HN had a CDKN2 mutation, which may be a positive biomarker for response to targeted CDK4/6 inhibitors in patients with advanced SC of HN. Moreover, they found that more than 50% of patients had at least one mutation in RTK such as EGFR, ALK, MET, suggesting that multiple targeting drugs approved may improve the long-term survival of patients with SC of HN. For example, a patient with SC of HN with an ALK translocation had stable disease for more than 4 months after treatment with crizotinib (20, 58).

In this case, the patient experienced a grade 2 adverse drug reaction of hypothyroidism during the course of treatment. As it was not possible to determine which specific medication caused the reaction, we decided to temporarily suspend the anti-tumor therapy and initiate thyroid hormone replacement therapy. After one month of treatment, the patient’s thyroid function recovered to a grade 1 level, and subsequently, we resumed the anti-tumor treatment. Hypothyroidism is one of the most common endocrine diseases in ICIs therapy, usually occur within 3 weeks to 10 months after the start of ICIs treatment. Studies have indicated that the incidence of hypothyroidism when using PD-1 inhibitors alone ranges from 3.9% to 8.5%, while it increases to 10.2% to 16.4% with combination ICIs therapy (59). It is important to note that our patient did not experience any severe immune-related adverse reactions aside from hypothyroidism. Common adverse effects associated with the use of anlotinib include hypertension, hand-foot syndrome, hypothyroidism, fatigue, and diarrhea (60). Yu et al. conducted a retrospective study showing that hypothyroidism and liver function abnormalities were the most frequently observed adverse reactions in patients receiving ICIs combined with anlotinib for small cell lung cancer treatment (61). In this case, the concurrent use of sintilimab and anlotinib seems to increase the risk of developing hypothyroidism. Therefore, we regularly monitor indicators such as liver function, kidney function, cardiac enzyme profile, thyroid function, adrenal cortex function, and blood pressure to prevent and promptly manage potential adverse reactions.

Overall, although previous studies have shown that the average survival of patients with advanced head and neck sarcomatoid carcinoma with recurrent metastases is in the range of 2-5 months, our patient achieved good local control after receiving first-line antitumor therapy with anlotinib in combination with sintilimab, and is still in PR. Therefore, based on this case and previous studies, we propose that antiangiogenic agents combined with ICIs may be an effective strategy for the treatment of patients with advanced sarcomatoid carcinoma of the head and neck. This regimen has the potential to significantly improve patients’ quality of life and prolong survival time. Of course, there are some limitations in this case. Firstly, only one patient was reported in this paper and the limited data failed to analyze the genetic mutation status of the patient. In the future, we need more large-scale randomized controlled trials to study the efficacy and safety of ICIs in patients with sarcomatoid carcinoma of the head and neck.

## Data availability statement

The original contributions presented in the study are included in the article/supplementary material. Further inquiries can be directed to the corresponding author.

## Ethics statement

Written informed consent was obtained from the individual(s) for the publication of any potentially identifiable images or data included in this article.

## Author contributions

LW: Writing – original draft, Writing – review & editing. YH: Writing – review & editing. XS: Writing – original draft, Writing – review & editing.
